# Nature-Based Relaxation Videos and Their Effect on Heart Rate Variability

**DOI:** 10.3389/fpsyg.2022.866682

**Published:** 2022-06-10

**Authors:** Annika B. E. Benz, Raphaela J. Gaertner, Maria Meier, Eva Unternaehrer, Simona Scharndke, Clara Jupe, Maya Wenzel, Ulrike U. Bentele, Stephanie J. Dimitroff, Bernadette F. Denk, Jens C. Pruessner

**Affiliations:** ^1^Department of Psychology, Division of Neuropsychology, University of Konstanz, Konstanz, Germany; ^2^Child and Adolescent Research Department, Psychiatric University Hospitals Basel (UPK), University of Basel, Basel, Switzerland

**Keywords:** nature video, relaxation, early life adversity, trait mindfulness, heart rate variability

## Abstract

Growing evidence suggests that natural environments – whether in outdoor or indoor settings – foster psychological health and physiological relaxation, indicated by increased wellbeing, reduced stress levels, and increased parasympathetic activity. Greater insight into differential psychological aspects modulating psychophysiological responses to nature-based relaxation videos could help understand modes of action and develop personalized relaxation interventions. We investigated heart rate variability (HRV) as an indicator of autonomic regulation, specifically parasympathetic activity, in response to a 10-min video intervention in two consecutive studies as well as heart rate (HR). We hypothesized that a nature-based relaxation video elicits HRV increase and HR decrease, with response magnitude being affected by aspects of early life adversity (conceptualized as low parental care and high overprotection/constraint) and trait mindfulness. In Study 1, *N* = 60 participants (52% female, age*_*mean*_* = 23.92 ± 3.13 years, age*_*range*_* = 18–34 years) watched a relaxation video intervention depicting different natural scenery. We analyzed changes in HR and respiratory sinus arrhythmia (RSA) as a standard HRV measure, both based on 3-min segments from the experimental session, in multiple growth curve models. We found a decrease in HR and increase of RSA during the video intervention. Higher paternal care and lower trait mindfulness observing skills (assessed via questionnaires) were associated with higher RSA values before but not during video exposure. In Study 2, *N* = 90 participants (50% female, age*_*mean*_* = 22.63 ± 4.57 years, age*_*range*_* = 18–49 years) were assigned to three video conditions: natural scenery from Study 1, meditation video, or short clip from “The Lord of the Rings.” Again, HR decreased, and RSA increased during video segments, yet without expected group differences across different video types. We found higher parental care and lower parental overprotection to predict higher RSA at different times during the experiment. Interestingly, lower paternal overprotection predicted overall higher RSA. These results suggest a generic relaxation effect of video interventions on autonomic regulation that we discuss in light of different theories mapping restorative effects of natural environments. Further, psychological characteristics like aspects of early life adversity and trait mindfulness could contribute to individual differences in autonomic regulation. This study contributes to a better understanding of autonomic and psychological responses to relaxation videos.

## Introduction

Relaxation videos gained in popularity not only on online streaming platforms like YouTube but in empirical research as well as indicated by a massive growth in publications related to the topic ‘‘relaxation video’’ over the past decades, with 8 publications listing ‘‘relaxation video’’ in the title in 1989, and 114 in 2020^1^. To understand the psychological and physiological effects of relaxation videos, standardized measures to identify the various aspects of reactivity to relaxation are needed (Meier et al., 2020). In addition to subjective changes in affect and arousal, relaxation triggers changes in the autonomic nervous system (Chang et al., 2011). It can be described as a decrease in physiological arousal (Smith, 2007). Various physiological markers have been used to assess the changes associated with relaxation, such as a decrease in blood pressure and heart rate (HR). However, taking a closer look at the autonomic nervous system, which regulates the stress and relaxation response, shows that it is the increased activity of the parasympathetic nervous system that is associated with relaxation (e.g., Bertsch et al., 2012). Parasympathetic activity is related to decreased cardiovascular, respiratory, and electrodermal activity (Kreibig, 2010) and increase in positive mood (Shaffer and Ginsberg, 2017). A reliable measurement for parasympathetic activity is a vagally mediated marker of heart rate variability (HRV), high-frequency HRV (HF-HRV), which corresponds with respiratory sinus arrhythmia (RSA) (Acharya et al., 2006). Therefore, in this study we will look at relaxation as the increase in parasympathetic activity as indicated by RSA changes. While HF-HRV and RSA are the most specific indicators of parasympathetic activity (Thayer et al., 2012), HR is frequently reported in relaxation research and thus included as an additional parameter in this study.

Previous studies suggest an effect of relaxation videos including natural environments on autonomic regulation. For example, participants watching 360° nature videos (Irish countryside or Australian beaches) after a cognitive stressor showed significant stronger physiological relaxation than the control group, who viewed a video of an empty classroom, indicated by a reduction in electrodermal activity and an increase in HRV (Anderson et al., 2017). This line of research builds on several theories that explain why nature environments have a restorative effect, associated with an increase in parasympathetic activity, positive mood and replenish resources. For one, the Biophilia Hypothesis (Kellert and Wilson, 1993) describes biophilia as an inherent longing for connection to other living things, including flora and fauna. Further, the Attention Restauration Theory (Kaplan, 1995) focuses on the aspects of nature that help with recovery from mental fatigue, stating that the effortless preference for natural scenes is the needed counterpole to the stressful challenges of everyday life. Finally, the Psychoevolutionary Theory (Ulrich, 1983) emphasizes the esthetic preference for natural scenes and focuses on the evolutionary advantage of a quicker recovery from stressful situations. Recovery involves a positive affective reaction with a decrease in negative emotions like fear and reduction of physiological arousal. This reaction can be seen, for example, in the effects of a short walk in a forest which has been shown to reduce blood pressure and HR, increase HF-HRV (Lee et al., 2014), increase positive affect (Payne and Delphinus, 2019) and decrease negative affect, e.g., anxiety (Song et al., 2018). This effect of real-life nature seems to translate to sole representations of nature, like pictures or videos of rivers, forests, or even fireplaces (Dana Lynn, 2014). Watching nature pictures increased HRV in a resting state (Gladwell et al., 2012) and in a recovery phase after a stressor (Brown et al., 2013) but watching urban pictures did not. This hints at nature exposure leading not only to benefits in recovery phases but as well having buffering effects (Beute and De Kort, 2014). However, not all studies come to this conclusion. Van den Berg et al. (2015) found a greater recovery after a stressor while watching nature pictures compared to urban pictures, but no buffering effect. A 2 h long forest bathing experience (Yu et al., 2017) and looking at fresh roses (Song et al., 2017) had no significant effect on parasympathetic activity measures by HF-HRV. One study even found an opposite effect: HRV (indicated by RSA) decreased and heart rate increased when exposed to real-life nature during a 5-day field trip (Scott et al., 2021). However, most of these studies used real-life nature stimuli. While it can be assumed that real-life and virtual nature stimuli might elicit comparable effects (Browning et al., 2020) real-life nature stimuli lack standardization capability and accessibility across different research sites and sample composition. Moreover, these mixed results show, that further studies are needed to investigate, why exposure to natural stimuli only sometimes has relaxing effects. Keeping especially virtual nature interventions in mind when investigating this question is important since, video-based nature interventions could help scientific studies as they are easier to implement and standardize than a real-life outdoor setting that is subject to, e.g., weather conditions.

Even though there are studies showing an increase in relaxation in reaction to either a real (e.g., Song et al., 2018) or virtual nature environment (e.g., Anderson et al., 2017) as described above, there is little research about individual predispositions influencing the relaxation response. A first review suggests that physiological relaxation (e.g., HRV, blood pressure) in reaction to indoor nature stimuli (e.g., pictures of forests, potted plants) is influenced by age, gender and personality (Jo et al., 2019). Jo et al. (2019) referred to interindividual differences in general, without taking a closer look at specific factors. One of the factors possibly influencing the relaxation response could be early life adversity, i.e., the experience of adverse and potentially harmful situations in childhood and adolescence, like poverty, neglect or child abuse (Smith and Pollak, 2021). Such experiences can influence the autonomic nervous system and lead to lower resting levels of HRV (Sigrist et al., 2020), which might moderate the influence of early life adversity as a risk factor for psychological disease (Shonkoff et al., 2012). Moreover, early life adversity could alter an organisms capability to relax and thereby counteract negative effects of stress (Bhasin et al., 2013) as well, which could play a role in the mechanisms behind early live adversity being a risk factor for psychopathology (Lima et al., 2010). This could, for example, be demonstrated through a decreased HF-HRV in reaction to a nature based relaxation intervention, as can be observed in depressed patients (Matsui et al., 2016). On the opposite endpoint of the physiological activation-deactivation continuum, early life adversity is a prominent research factor that is assumed to alter the psychophysiological stress response (Tarullo and Gunnar, 2006). It is often operationalized as a lack of parental care (Luecken, 2000; Engert et al., 2010).

In contrast, trait mindfulness, i.e., the general ability of being aware of one’s present surroundings, emotions and thoughts, is associated with life satisfaction, optimism and mental health (Brown and Ryan, 2003) and could thus positively contribute to the relaxation response. It is part of a mindfulness practice to concentrate on what one can see and hear, without letting the thoughts wander to different, potentially stressful topics. Therefore, trait mindfulness could be an advantage when exposed to relaxation videos and increase their effects on the parasympathetic nervous system.

In two independent experiments, we aimed to investigate autonomic responses to different video contents and their generic ability to induce physiological relaxation in two consecutive studies, while exploring the potential impact of interindividual differences. In Study 1, we investigated whether popular nature-based videos (including audio) showing either a river stream in a forest, rain in the thicket, the crackling of burning wood in a fireplace, or waves crashing on a beach induce a psychophysiological relaxation response. We hypothesized that HR decreases and HRV increases as a result of the (video-based) nature exposure alone in comparison to a questionnaire baseline controlling for sex, age, and depressive symptoms (cf. Laborde, 2017). Building on the results of Study 1, we conducted Study 2 to extend the findings by adding a physiological baseline and two more video conditions: a guided meditation video and a control condition using a movie clip of “The Lord of the Rings.” In Study 2, we hypothesized that HR decreases and HRV increases during the nature videos and during the guided meditation video but not during the movie clip. In addition to the effect of different video contents, in both studies we investigated the potential influence of two psychological factors that might modulate the relaxation response: early life adversity as a risk factor for psychopathology and trait mindfulness as a protective factor associated with metal health. We thereby aimed to investigate their influence on relaxation as proxies for psychological or personality-related factors as reported by Jo et al. (2019). We hypothesized that early life adversity would lead to less increase of HRV values in reaction to the relaxation videos and trait mindfulness would increase the relaxing effects of those videos, leading to stronger increases of HRV.

## Study 1

### Method

#### Participants

In Study 1, we assessed *N* = 60 students (sex assigned at birth: 52% female, 48% male; age: *mean* = 23.92 ± 3.13 years, range 18–34 years) who responded to our advertisements posted on site at the University of Konstanz (flyers and online participant recruitment platform of the University of Konstanz). For inclusion in the study, participants were required to be at least 18 years of age, have sufficient German language skills, and be free of cardiovascular diseases (e.g., coronary heart disease or heart implants) that could influence HRV measures. Inclusion criteria were part of the advertisements and verified during the laboratory assessment. Data collection for Study 1 took place from April to May 2018. All participants received 10€ or 1 h of course credit as study compensation after debriefing. The study protocol was approved by the Ethics Committee of the University of Konstanz and followed the guidelines outlined in the Declaration of Helsinki. Sample size was determined using the tool G*Power (Faul et al., 2007) based on within-between interactions (two groups, male and female participants, and four time points) and medium effect size, power = 90%, alpha = 0.05 as well as feasibility considerations and previous studies on HRV reactivity (Gladwell et al., 2012).

#### Procedure

**FIGURE 1:**
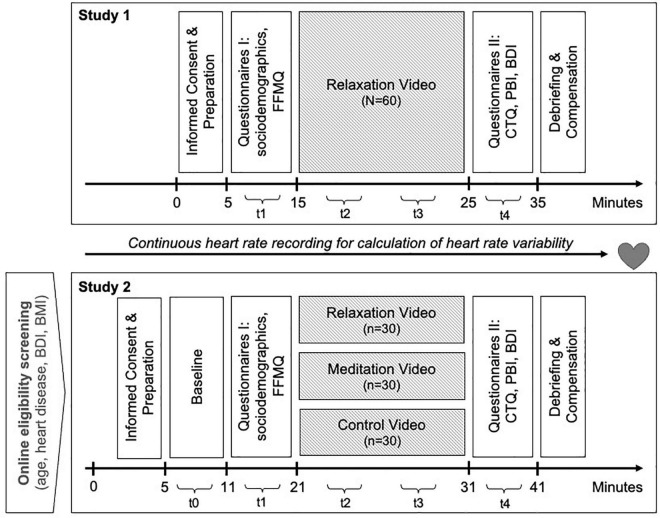
Schematic representation of the study design for study 1 and 2; t0 – t4: 180-sec time segments that were extracted from the continuous heart rate recording for calculation of heart rate and heart rate variability resulting in four time points for the multiple growth curve analysis in Study 1 and five time points in Study 2. A comprehensive list of all measures assessed during both studies can be found at https://osf.io/wcdku/.

After providing informed consent and applying the HRV sensors, participants completed a first set of paper and pencil questionnaires, assessing sociodemographic information and trait mindfulness. Subsequently, we presented all participants with the relaxation video intervention, consisting of one of the relaxation videos described below. Finally, participants completed a second set of questionnaires, including measures of early life adversity and depressive symptoms. We split all questionnaires into the two sets to be minimally arousing before the relaxation video intervention. Laboratory assessments took place between 8.00 a.m. to 1.15 p.m. in a windowless room. An overview of the procedure of both studies is depicted in Figure 1.

#### Video Material

For the 10-min video intervention, participants chose two out of four relaxation themes based on personal preference to avoid aversive associations with one of the themes: (1) river stream in the forest and bird sounds (Lawson, 2013), (2) rain falling in the thicket (Paunchev, 2017), (3) sunny tropical beach (Outstanding Videos, 2012), and (4) fireplace (Balu, 2017). We selected these relaxation video clips based on the YouTube search terms “relaxation videos,” “calm nature,” and “relaxation” and their respective number of views. Each clip had more than two million views by March 2018 and was presented on a 17-inch ThinkPad (Lenovo, Hongkong, China). We took 5 min from each of the videos and merged them to avoid distraction during clip transition. Using circumaural earphones (Sennheiser, Wedemark, Germany), we tried to maximize video audio and minimize ambient noise.

#### Measurements

##### Heart Rate Variability

To investigate autonomic regulation during the different experimental phases, we recorded HR continuously using the Polar H7 heart rate sensors (Polar Electro Oy, Kempele, Finland) on a two-electrode chest belt. The HR sensors were connected via Bluetooth with an 11-inch iPad (Apple Computer, Cupertino, CA, United States) running the HRV Logger App for iOS (Altini, 2013) that records and stores HR and beat-to-beat intervals (RR-intervals) at a sampling rate of 1000 Hz together with event markers, set manually throughout the session to allow separating the experimental phases. After transferring data to local desktop computers in the lab, we processed RR data using R (version 4.1.0) together with its user interface R-Studio (version 1.4.1717) to remove ectopic beats, artifacts and interpolate missing beats based on visual inspection and facilitated by in-house R scripts^2^. As it is recommend to calculate HRV in constant time intervals lasting between 1 and 5 min (Laborde, 2017), we subsequently extracted 180-s segments, each from the middle of the respective experimental phases: one segment from each of the two sets of questionnaires before (t1) and after the video (t4) and two segments from the video section (t3 and t4), which we split in half before segment extraction to maximize information gain. Finally, using the R package RHRV (García Martínez et al., 2017), we computed RSA, in the context of this study defined as the natural logarithm of HF-HRV. For the calculation of HF-HRV in the fixed frequency bandwidth of 0.15–0.4 Hz (Shaffer and Ginsberg, 2017), we applied the frequency analysis provided by RHRV with a Fast Fourier Transformation using a window of 60 s and a shift of 30 s (Protocol and R scripts see text footnote 2).

##### Early Life Adversity

We retrospectively assessed self-reported early life adversity with the Parental Bonding Instrument (PBI, Parker et al., 1979). The PBI focuses on attachment aspects of perceived parenting behavior which can be considered one type of early life adversity if it is disadvantageous (lack of care and/or high overprotection and constraint) (Unternaehrer et al., 2021). Participants rate perceived parenting behavior of their mothers and fathers respectively on the two dimensions care and overprotection with 25 Items per parent on a 5-point Likert-Scale. Higher sum scores on the care dimension indicate warm and thoughtful parenting and are associated with beneficial parenting effects. Higher scores on the overprotection dimension indicate controlling and restrictive parenting and are associated with disadvantageous parenting effects. For this study we used a German translation of the PBI that was not validated when we conducted the study (Lutz et al., 1995). However, a very similar version has since been validated by our group, demonstrating good psychometric properties (Benz et al., 2021).

##### Trait Mindfulness

We used the Five Facet Mindfulness questionnaire (FFMQ, Baer et al., 2006) to measure trait mindfulness, the general ability to deliberately direct one’s attention toward the present moment without judgment or immediate reaction. In the FFMQ, participants rate their trait mindfulness via 39 items on a 5-point Likert-Scale. As the FFMQ describes trait mindfulness as a multifaceted construct, answers can be grouped to different mindfulness skills on five subscales: observing, describing, non-reacting, non-judging, acting with awareness. The German version of the FFMQ showed good reliability and validity (Michalak et al., 2016). Additionally, we asked participants if they had any previous experience with mindfulness-based practices (e.g., yoga or meditation practice or a structured course program) and if so, for how many years and how regularly they engage in mindfulness practices.

##### Depressive Symptoms

Using the Beck’s Depression Inventory II (BDI-II, Hautzinger et al., 2006), we assessed depressive symptoms, which has been associated with HRV previously (Kemp et al., 2010) and thus included as a potential covariate. The BDI-II entails different symptoms of a depressive episode according to the ICD-10 (Graubner, 2013), with a total of 21 questions answered on a 4-point Likert-Scale with verbal anchor descriptions.

#### Statistical Analysis

Before statistical analysis, we checked for outliers in HR and RSA and winsorized values that lay more than three standard deviations above or below the mean across all conditions and timepoints. Missing items in one of the questionnaires were imputed with the item median (*k* = 13 missing items in the PBI, *k* = 7 missing items in the FFMQ). If a participant omitted more than 20% of the items of a given subscale, this variable was coded as NA and excluded from the respective analysis (compare Table 1).

**TABLE 1:** Sample characteristics in Study 1.

	Study 1
	
	Relaxation (*N* = 60)
Sex (% female)^a^	51.7%
Age (in years)	23.92 (±3.13)
Job (% students)	100%
BDI-II	9.78 (±8.75)
*Mindfulness experience*	
Any experience	36.7%
Years of experience^b^	2.88 (±2.50)
Regularity of practice^b^ (% at least weekly)	20%
*FFMQ*	
Total Score	3.44 (±0.42)
Observing	3.48 (±0.65)
Describing	3.55 (±0.69)
Non-judging	3.73 (±0.77)
Non-reacting	3.09 (±0.58)
Acting with awareness	3.33 (±0.64)
*PBI mother*	
Maternal care	27.03 (±6.70)
Maternal overprotection	12.22 (±7.70)
*Father* (*n* = 56)^c^	
Paternal care	24.61 (±7.85)
Paternal overprotection	7.54 (±5.73)

*^a^Self-reported sex as assigned at birth, with the response options “female,” “male,” “diverse,” n = 0 answered “diverse,” thus only two categories (female/male) are reported.*

*^b^Based on n = 24 participants with mindfulness experience only.*

*^c^Reduced sample sizes are due to missing items as described in the “Method” section; BDI-II, Beck Depression Inventory; FFMQ, Five Facet Mindfulness Questionnaire; PBI, Parental Bonding Instrument.*

To run our analyses, we used R version 4.1.0 (R Core Team, 2021), RStudio version 1.1.463 (RStudio Team, 2016), and the packages “arsenal” (Heinzen et al., 2021) for descriptive analyses and “nlme” (Pinheiro et al., 2021) for mixed model analyses. Graphs were created using “ggplot2” (Wickham, 2016). The level of significance was set to an alpha level of 0.05, and Bonferroni corrections were applied as necessary.

In a first step, we used multilevel mixed models to calculate growth curve analyses to model trajectories for HR and RSA over time. In multilevel models one can account for individual differences in average HR and RSA levels as well as different time trajectories (change over time). If addition of a random effect significantly improved model fit, we assumed that there was a significant amount of variance explained by inter-individual differences, either regarding average outcome values (intercept) or regarding change in outcome values over time (slope). The analysis of Study 1 included four time points: first set of questionnaires (t1), first half of videos (t2), second half of videos (t3), and second set of questionnaires (t4). We first defined the basic model structure regarding fixed and random effects by comparing model fit indices to find the model that best explained the data using LogLikelihood ratio tests. If the addition of the respective step improved the model fit significantly, this change was retained for the next step. We first introduced a fixed intercept, then random intercept on participant level, followed by fixed time effects for linear, quadratic and cubic trends over time (each evaluated one after the other), and finally random effects of the best-fitting time trend. To this basic model, we added potential covariates (sex, age, and BDI-II) one at a time to examine their effects on HR or RSA. Finally, we investigated the influence of the secondary predictors, early life adversity and trait mindfulness by adding each of the four PBI subscales (maternal and paternal care and overprotection) and each of the five FFMQ subscales (observing, describing, non-reacting, non-judging, acting with awareness) individually as interaction with trend of time to the final model. For these secondary predictors, we applied Bonferroni corrections for *k* = 4 or *k* = 5 tests, respectively, thus adjusting the level of significance to α_Bonferroni_ = α/k.

Using the R package “performance” (Lüdecke et al., 2021), we checked model assumptions and performance including the function R2() to evaluate effect size for all final models. An R Markdown file with the statistical analysis can be found at https://osf.io/wcdku/.

### Results

#### Sample Characteristics

We assessed *N* = 60 students in Study 1. Table 1 displays the demographic and psychometric characteristics of both samples and the different video conditions. Regarding nature video content, we found that most participants chose “rain falling in the thicket” (36%); followed by “river stream in the forest” (31%), “beach” (23%), and “fireplace” (11%). Due to the unbalanced distribution of the selected video contents, this factor was not included in the subsequent analysis.

#### Heart Rate and Heart Rate Variability

**FIGURE 2:**
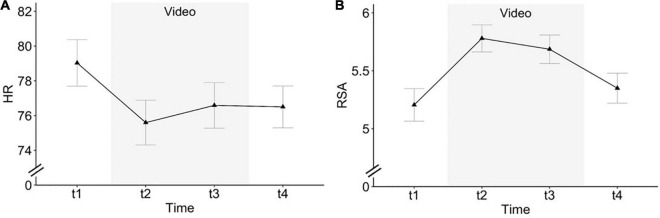
**(A)** Heart rate (HR) and **(B)** respiratory sinus arrhythmia (RSA) over time in Study 1 (t1: first set of questionnaires, t2: first half of video, t3: second half of video, t4: second set of questionnaires); error bars depict standard errors of the mean.

In Study 1, we analyzed HR and RSA over four time points (t1: first set of questionnaires, t2: first half of videos, t3: second half of videos, and t4: second set of questionnaires) looking at the effect of the nature-based relaxation videos in contrast to the two sets of questionnaires before and after the video. We found a significant decrease of HR and increase of RSA, respectively, while watching the video (see Figures 2A,B). For HR, in a mixed model with random intercept (Intra-Class Correlation Coefficient (ICC) = 0.90) and fixed slope, the model including a cubic trend of time (omnibus test: *F*(3,177) = 16.14, *p* < 0.001, marginal *R*^2^ = 0.17) to predict HR decrease during the video segments showed the best fit. For HRV, the best model fit was achieved by a random intercept (ICC = 0.74) and random slope for the linear time trend in combination with a fixed quadratic effect of time (omnibus test: *F*(2,178) = 33.80, *p* < 0.001, conditional *R*^2^ = 0.84, marginal *R*^2^ = 0.05) predicting RSA increase during the video segments. Potential covariates (age, sex, and BDI-II) did not change the results nor improve model fit for HR or RSA and were thus not included in the final model (see Table 2).

**TABLE 2:** Summary of final model to predict HR and HRV in Study 1.

Effects	Statistics	*P*-value
**HR**		
Intercept	*F*(1,177) = 3810.43	<0.001
Time^3^	*F*(3,177) = 16.14	<0.001
**HRV**		
Intercept	*F*(1,178) = 2231.60	<0.001
Time^2^	*F*(2,178) = 33.80	<0.001

*Linear mixed-effects model fit by maximum likelihood; HR-Model with random intercept and fixed cubic effect of time; HRV-Model with random intercept and fixed and random quadratic effect of time; HR, Heart Rate; HRV, Heart Rate Variability.*

#### Early Life Adversity and Trait Mindfulness

Adding PBI subscales as secondary predictors to the mixed model regarding HR, we found a significant interaction for the quadratic time effect with maternal care (model coefficients: estimate = –0.92, se = 0.42, *t*(174) = –2.19, *p* = 0.030) and paternal care (model coefficients: estimate = 0.79, se = 0.35, *t*(162) = 2.25, *p* = 0.026). Higher maternal care was associated with lower HR before and during the first half of the video (t1 and t2); higher paternal care was associated with lower HR during the first half of the video only (t2). For RSA, only the interaction of quadratic time and paternal care reached significance (mode coefficients: estimate = 0.15, se = 0.05, *t*(164) = 2.81, *p* = 0.006) participants with higher paternal care showing higher RSA before and after (t1, t4) but not during the video (t2, t3). However, after Bonferroni correction for four tests (α_Bonferroni_ = 0.013), only the interaction effect of paternal care and quadratic time to predict RSA remained significant.

Concerning trait mindfulness and HR, we found an interaction between quadratic time and FFMQ-observing (model coefficients: estimate = 11.6, se = 4.27, *t*(174) = 2.71, *p* = 0.007). For RSA, we found an interaction between the quadratic time trend and FFMQ-observing (model coefficients: estimate = –1.90, se = 0.65, *t*(176) = –2.91, *p* = 0.004). Both effects of FFMQ-observing skills survived Bonferroni correction for five tests (α_Bonferroni_ = 0.01). Higher FFMQ-observing scores were associated with higher HR and lower RSA before (t1) but not during the video segment (t2, t3).

### Discussion

In Study 1, we investigated changes of HR and RSA as indicators of autonomic regulation in response to a nature-based relaxation video intervention. During the video, HR decreased, and RSA increased in line with our hypotheses in female and male participants alike. Additionally, we observed lower RSA in participants with low paternal car and high mindfulness observing skills with the latter contradicting to our expectations. These results suggest a positive effect of the nature-based relaxation video intervention on autonomic regulation. However, some methodological limitations should be considered in the interpretation of these observations especially regarding the secondary predictors, early life adversity and trait mindfulness. First of all, power analysis for this was based on a comparison of HR and RSA changes across four time points between female and male participants. Thus, the sample size might have been too small to detect effects of the secondary predictors and extension and replication of these findings in bigger samples is needed. In addition, only few exclusion criteria were applied here. Even though the BDI-score did not influence the present results, many studies reported a negative effect of depressive symptoms and other factors on HRV (Kemp et al., 2010). A more rigorous sampling that accounts for effects of depressiveness, and under- or overweight (Peyser et al., 2021; Strüven et al., 2021) could benefit future studies. Furthermore, this study only looked at one video condition in comparison to questionnaires before and after the videos. It would be interesting to include a resting-state baseline (Laborde, 2017) and compare the nature-based relaxation videos to other video types. Finally, we did not assess any subjective measures on feelings of relaxation after watching the video or whether a participant was familiar with the video content beforehand. For example, it might be possible, that one of the videos being someone’s favorite relaxation video could have influenced the results.

## Study 2

Based on the observed effect of the nature-based relaxation videos on autonomic regulation in Study 1 and the partial modulation by aspects of early life adversity and trait mindfulness, we aimed to replicate and extent this finding in an independent sample in Study 2. In addition to employing more rigorous exclusion criteria and a physiological baseline, we added two video conditions to compare the nature-based relaxation videos from Study 1 with other video types: a meditation video, as a different approach to relaxation via inducing mindfulness (Jain et al., 2007), and a movie clip from “The Lord of the Rings,” as a control condition that is attention grabbing without aiming at relaxation (Zeidan et al., 2010).

### Method

#### Participants

Data collection for Study 2 took place from October to November 2018. In addition to the exclusion criteria used in Study 1, we used an online pre-screening to exclude interested students if they reported being under- or overweight, i.e., a body mass index (BMI) < 17.5 or > 30, or having clinically relevant depressive symptoms, i.e., a BDI-II score > 18. A total of *N* = 90 students (sex assigned at birth: 50% female, 50% male; age: *mean* = 22.63 ± 4.57, range 18–49) participated in the study. Sample size was determined using the tool G*Power (Faul et al., 2007) based on within-between interactions (three video conditions groups and four time points) and medium effect size, power = 90%, alpha = 0.05 as well as feasibility considerations and previous studies on HRV reactivity (e.g., Gladwell et al., 2012).

#### Procedure

The laboratory assessment in Study 2 resembled Study 1 if not described otherwise (see Figure 1). After giving their informed consent and applying the HRV sensor, participants first underwent a 6-min physiological baseline (resting-state, eyes open, sitting upright with both feet on the ground) before completing the first set of questionnaires. Subsequently, all participants completed the video intervention. In Study 2, we created a quasi-randomization of subject to video condition by generating a random condition-by-sex list using the R sample function to distribute group assignment randomly across experimenter, day, time and participantś sex. This way, we assigned participants to one of three video conditions: the same nature-based relaxation video intervention as in Study 1, a meditation video, or a control video (see below). For all three conditions, they were instructed to concentrate on the video without mentioning the word “relaxation.” Finally, participants completed the second set of questionnaires. Additionally, we asked them to rate the videos on a 5-point Likert scale with respect to familiarity, pleasantness, and subjective relaxation effect of the videos. Laboratory sessions were scheduled between 8 a.m. and 7 p.m. in empty classrooms at the University of Konstanz, where we seated participants facing a white wall.

#### Video Material

In addition to the nature-based relaxation videos (describe above), two more video conditions were added to the design in Study 2. Participants watched the 10-min videos on a 13-inch MacBook Air (Apple Computer, Cupertino, CA, United States) and used circumaural earphones (Sennheiser, Wedemark, Germany) to maximize video audio and minimize ambient noise.

The meditation video used in Study 2 invited participants to practice a beginner’s mindfulness exercise guided by a female voice (Morrison, 2017). Meditation instructions included the two-fold observation of breathing and thoughts without analyzing, judging, or changing them. For comparability to the relaxation group, we combined the meditation instructions with the beach scenery (Outstanding Videos, 2012) and calm, melodic music.

As a control condition, we presented the third group in Study 2 with a film clip from the movie “The Lord of the Rings: The Fellowship of the Ring” (Jackson, 2001, min[6.40–16:40]), specifically with the arrival of Gandalf, a wizard and main character of the movie, at the Shire, a hilly green inland area. We aligned this control condition with other meditation studies, that used an audio segment of the same movie series for their control condition (Zeidan et al., 2010). In addition, we expected this film clip to resemble the relaxation group in the general appearance (naturalistic landscape, no distressing or startling aspects) but without the relaxation focus.

#### Measurements

The measurements used in Study 2 to assess HRV, early life adversity, trait mindfulness, and depressive symptoms resembled Study 1 except two modifications: First, the physiological baseline implemented in Study 2 before the first set of questionnaires was added as the first time point (t0). To analyze this segment, we extracted one 180-s RR-interval from the middle of the 6-min baseline. Second, we used a BDI-II score > 18 in a pre-screening as an exclusion criterion for the laboratory assessment.

#### Statistical Analysis

We applied the same pre-processing rational to outliers and missing values and the same growth curve analysis procedure as in Study 1. The available HRV time intervals entering into the analysis were: physiological baseline (t0), first set of questionnaires (t1), first half of videos (t2), second half of videos (t3), and second set of questionnaires (t4). After exploring random effects for intercept (outcome value) and slope (time variable) in addition to a linear, quadratic, and cubic time trend and adding potential covariates (sex, age, BDI-II, and mindfulness experience) as described for Study 1, we examined the main effects of video condition and the interaction between video condition and the time trends on HR or RSA. Finally, as in Study 1, we investigated the influence of the secondary predictors, early life adversity and trait mindfulness by adding each of the four PBI subscales (maternal and paternal care and overprotection) and each of the five FFMQ subscales (observing, describing, non-reacting, non-judging, acting with awareness) individually as interaction with trends of time to the final model. For these secondary predictors, we applied Bonferroni corrections for *k* = 4 or *k* = 5 tests, respectively, thus adjusting the level of significance to α_Bonferroni_ = a/k. Again, for every step of model building, resulting model fits were evaluated using the Log-Likelihood Ratio as described above. An R Markdown file with the statistical analyses of Study 1 and study 2 can be found at https://osf.io/wcdku/.

### Results

#### Sample Characteristics

In Study 2, we assessed *N* = 90 people. Table 3 displays the demographic and psychometric characteristics of the three video condition groups (*n* = 30 each). Except for mindfulness experience the groups did not differ in any of these characteristics. Furthermore, the movie clip was rated as significantly more familiar, while all three videos were mostly rated as pleasant and relaxing. Regarding nature video content, we found that most participants chose “rain falling in the thicket” (32%); followed by “river stream in the forest” (27%), “fireplace” (22%), and “beach” (20%). Due to the unbalanced distribution of the selected video contents, this factor was not included in the subsequent analysis.

**TABLE 3:** Sample characteristics and group comparison in Study 2.

	Study 2				
	
	Control (*n* = 30)	Meditation (*n* = 30)	Relaxation (*n* = 30)	*P*-value
Sex (% female) ^a^	50%	50%	50%	> 0.999
Age (in years)	22.43 (±3.28)	23.07 (±5.87)	22.40 (±4.22)	0.820
BMI	22.03 (±2.13)	22.23 (±2.69)	22.80 (±2.77)	0.483
Job (% students)	90%	86.6%	90%	0.513
BDI-II	5.97 (±4.61)	5.97 (±4.65)	5.80 (±5.05)	0.988
***Mindfulness experience***				
Any experience	**53.3%**	**50%**	**23.3%**	**0.036**
Years of experience ^b^	4.31 (±3.38)	4.69 (±4.80)	2.29 (±0.95)	0.369
Regularity of practice ^b^ (% at least weekly)	20%	23.3%	13.3%	0.600
***Video ratings***				
Familiarity ^c^	**3.87 (**±**1.07)**	**2.87 (**±**1.04)**	**2.67 (**±**1.24)**	**<0.001**
Pleasantness ^d^	1.73 (±0.87)	1.33 (±0.55)	1.73 (±0.91)	0.083
Feeling relaxed ^e^	1.93 (±0.87)	1.60 (±0.77)	1.87 (±0.94)	0.289
***FFMQ***				
Total Score	3.47 (±0.37)	3.41 (±0.37)	3.34 (±0.48)	0.515
Observing	3.40 (±0.56)	3.44 (±0.63)	3.54 (±0.50)	0.587
Describing	3.56 (±0.70)	3.47 (±0.60)	3.59 (±0.77)	0.777
Non-judging	3.73 (±0.76)	3.73 (±0.61)	3.34 (±0.83)	0.063
Non-reacting	3.21 (±0.59)	3.18 (±0.75)	3.13 (±0.68)	0.916
Acting with awareness	3.44 (±0.60)	3.20 (±0.56)	3.13 (±0.59)	0.105
***PBI Mother* (*n* = 89)**				
Maternal care	27.83 (±5.60)	28.57 (±5.16)	26.52 (±7.83)	0.452
Maternal overprotection	11.10 (±4.85)	9.2 (±5.99)	11.52 (±6.18)	0.253
*Father* (*n* = 85)				
Paternal care	23.54 (±7.21)	24.33 (±7.25)	22.60 (±6.60)	0.648
Paternal overprotection	7.86 (±4.42)	7.85 (±5.86)	8.90 (±6.77)	0.730

*P-values are reported for group comparisons based on Chi^2^-Tests or ANOVAs depending on data properties; significant group differences at a level of α < 0.05 are printed in bold; if not indicated otherwise the total sample of N = 90 participants was included in the comparison between the three groups, reduced sample sizes are due to missing items as described in the “Method” section.*

*^a^Self-reported sex as assigned at birth, with the response options “female,” “male,” “diverse,” n = 0 answered “diverse,” thus only two categories (female/male) are reported.*

*^b^Based on n = 36 participants with mindfulness experience only.*

*^c^Video familiarity rated on a 5-point Likert-Scale from 1 = very unfamiliar to 5 = very familiar.*

*^d^Video pleasantness rated on a 5-point Likert-Scale from 1 = very pleasant to 5 = very unpleasant.*

*^e^Feeling of relaxation after video rated on a 5-point Likert-Scale from 1 = very relaxed to 5 = not relaxed at all; BMI, Body Mass Index; BDI-II, Beck Depression Inventory; FFMQ, Five Facet Mindfulness Questionnaire; PBI, Parental Bonding Instrument.*

#### Heart Rate and Heart Rate variability

**FIGURE 3:**
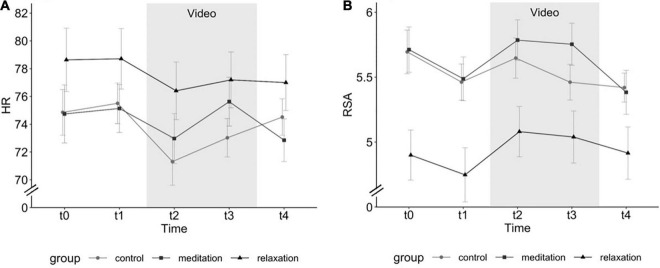
**(A)** Heart rate (HR) and **(B)** respiratory sinus arrhythmia (RSA) over time in study 2 (t0: baseline, t1: first set of questionnaires, t2: first half of video, t3: second half of video, t4: second set of questionnaires) in all three groups: nature-based relaxation video, meditation video, control video; error bars depict standard errors of the mean.

In Study 2, we compared three different video interventions at five time points (t0: baseline, t1: first set of questionnaires, t2/t3: two video segments, t4: second set of questionnaires). Comparable to Study 1, we found a significant decrease in HR and increase in RSA during the video segment compared to baseline (see Figures 3A,B). For HR, in a mixed model with random intercept (ICC = 0.89) and random slope, the quadratic term of time (omnibus test: *F*(2,354) = 2.53, *p* = 0.041) and the interaction effect between group and quadratic time (omnibus test: *F*(2,354) = 7.91, *p* < 0.001, marginal *R*^2^ = 0.03, conditional *R*^2^ = 0.93) significantly predicted decrease of HR during the video segment. The interaction effect of group with the quadratic time trend was mainly driven by a stronger decrease of HR during the video segment in the meditation group (model coefficients: estimate = –23.13, se = 7.44, *t*(354) = –3.11, *p* = 0.002). For HRV, the mixed model with random intercept (ICC = 0.76) and fixed slope controlled for age as a covariate (omnibus test: *F*(1,86) = 15.32, *p* < 0.001) the cubic effect of time (omnibus test: *F*(3,357) = 5.92, *p* < 0.001) showed a main effect of group (omnibus test: *F*(2,68) = 7.07, *p* = 0.001, conditional *R*^2^ = 0.78, marginal *R*^2^ = 0.21) predicting RSA increase during the video segment. Notably, the relaxation video group presented a reduced RSA throughout the experimental session independent of time (model coefficients: estimate = –0.60, se = 0.20, *t*(86) = –3.03, *p* = 0.003). As expected, higher age was associated with lower RSA. Other potential covariates (sex, BDI-II, mindfulness experience, and video familiarity) did not improve model fit neither for HR nor for RSA and were thus not included in the final model (see Table 4).

**TABLE 4:** Summary of final model to predict HR and HRV in Study 2.

Effects	Statistics	*P*-value
**HR**		
Intercept	*F*(1,354) = 6528.15	<0.001
Time^2^	*F*(2,354) = 7.91	<0.001
Video condition	*F*(2,87) = 1.19	0.308
Time^2^ x Video condition	*F*(4,354) = 2.53	0.041
**HRV**		
Intercept	*F*(1,357) = 4355.38	<0.001
Time^3^	*F*(3,357) = 5.92	0.001
Video condition	*F*(2,86) = 7.07	0.001
Age	*F*(1,86) = 15.32	<0.001

*Linear mixed-effects model fit by maximum likelihood; HR-Model with random intercept and fixed and random quadratic effect of time including the main and interaction effect of video condition; HRV-Model with random intercept and fixed cubic effect of time including age as a covariate and the main effect of video condition; HR, Heart Rate; HRV, Heart Rate Variability.*

#### Early Life Adversity and Trait Mindfulness

When adding PBI subscales as secondary predictors to the mixed models regarding RSA, we found a significant interaction for the quadratic time effect with maternal care (model coefficients: estimate = –0.4, se = 0.07, *t*(350) = –3.24, *p* = 0.001) and overprotection (model coefficients: estimate = 0.18, se = 0.08, *t*(350) = 2.20, *p* = 0.028). Higher maternal care and lower maternal overprotection predicted a more pronounced increase of RSA during the video segment (t2). In addition, we found a main effect of paternal overprotection, with higher overprotection predicting lower RSA throughout the experiment (model coefficients: estimate = –0.03, se = 0.02, *t*(80) = –2.09, *p* = 0.040). Only the interaction of maternal care and quadratic time to predict RSA survived Bonferroni correction for four tests (α_Bonferroni_ = 0.013). None of the PBI subscales significantly predicted HR. Concerning trait mindfulness, none of the FFMQ subscales revealed significant results when added to the models predicting HR or RSA.

### Discussion

In Study 2, we investigated changes of HR and RSA in response to three different video conditions, a nature-based relaxation video intervention, a meditation video, and a control movie clip. During all three video interventions, HR decreased, and RSA increased in line with our hypotheses. Looking at group effects, we observed a stronger RSA increase in the meditation group, and higher HR and lower RSA in the relaxation group throughout the whole experimental session. Mindfulness practices have been shown to elicit an increase in HRV (Azam et al., 2015), thus, the interaction effect for the meditation video is in line with our hypothesis and previous research. On the other side, the main effect of the relaxation group was more surprising. As this higher HR and lower RSA was present in this group even before the video intervention, it is likely not related to video characteristics but rather to group differences that might explain baseline differences in RSA. When comparing the relaxation group in Study 2 with the other groups, significantly less mindfulness experience stood out. Mindfulness expertise (Burg et al., 2012) has been linked to autonomic regulation, and HRV in particular, previously and, thus, might have contributed to lower RSA observed in this group. Yet, a number of other variables that were not measured in our study could play an important role as well, such as menstrual cycle, physical fitness, or psychopathology other than depression, which we could only speculate about.

Additionally, we observed higher RSA in participants with higher maternal car during the videos as expected. Overall, the results of Study 2 suggest a more general relaxation effect of all three video intervention on autonomic regulation.

## General Discussion

To investigate the effect of a nature-based relaxation video intervention on autonomic regulation, we measured HR and RSA throughout a 10-min video intervention in two consecutive studies. As intervention material, we used a nature-based relaxation video in Study 1 and compared this relaxation video to a meditation video and a control movie clip in Study 2. Both studies found an increase in RSA and a decrease in HR during the video segments in relation to the questionnaires before and after the video in the two independent samples as expected. However, contrary to our hypothesis on group differences in Study 2, the only interaction effect that we observed indicated a stronger HR decrease during the videos in the meditation video group.

The overall decrease in HR and increase in RSA in response to the videos suggests a successful activation of the parasympathetic system as a result of video exposure (Acharya et al., 2006; Shaffer and Ginsberg, 2017) in general. This is backed by the majority of subjective rating of the videos as pleasant and relaxing. Thus, the focus of the general discussion shifts from nature-based relaxation videos to relaxation videos in general. Our findings are in line with recent empirical evidence on the effects of relaxation videos. For example, watching videos at the workplace is helpful to recover from the stress and demands of work, increasing wellbeing, work satisfaction and relaxation (Janicke et al., 2018). Additionally, a video-based relaxation program successfully reduced anxiety, depressive and somatic symptoms in elderly participants with an anxiety disorder (Gould et al., 2019). Because of this and the convenience to standardize video interventions, they appear as a seminal method to induce relaxation in research settings and psychotherapy. Beyond that, experiencing relaxation is beneficial and linked to wellbeing and mental and physical health (Yu et al., 2010). However, standardized protocols with adequate control conditions and longitudinal investigations of the long-term effects of relaxation videos are missing.

While we hypothesized that nature and meditation videos would lead to a stronger relaxation response than the movie clip, we found a general response to any video that participants watched in both of our studies. First and foremost, this could be a general effect of sustained attention to a pleasant and non-threatening video stimulus. In contrast to the physiological baseline, looking at a blank classroom wall, or the questionnaire phases, looking down on a paper with the different questionnaires, the video intervention required participants to direct their attention to the screen which by itself might affect autonomic regulation (Holzman and Bridgett, 2017). Looking closer at movie clip content reveals another possible explanation for the comparable increase in RSA in this condition. The movie scene from “The Fellowship of the Ring” is set in the Shire with green hills, widespread meadows, and a blue sky underpinned with beautiful calm music. The contents are similar to those of the relaxation videos: a calm nature scene accompanied with instrumental music. When planning this study, we attempted to align the control condition as much as possible with the relaxation video, e.g., in colors and general atmosphere, yet without the specific aim of a relaxation video but with a distracting movie content similar to other studies (Zeidan et al., 2010). However, these features that made the movie clip comparable to the nature relaxation videos might have induced a comparable level of relaxation in the control condition compared to the other videos. According to the *Attention Restoration Theory* (ART, Kaplan, 1995), an environment needs four different aspects to trigger relaxation: *Fascination*, an environment’s ability to capture involuntary attention, *Being Away*, a physical and mental distance to everyday life, *Extent*, the sufficiency of an environment to engage the viewer’s mind, and *Compatibility*, the fit between the viewers’ intentions and the possibilities the environment offers. All four aspects of the ART can be identified in the nature-based relaxation videos, the meditation video, and the movie clip alike, giving a possible explanation of why RSA as an index of relaxation increased in all conditions. As such, our choice of a control condition using this particular excerpt from the movie was perhaps not a control condition, but a relaxing intervention as well.

After analyzing the effect of the different video conditions on autonomic regulation, we included parental care and overprotection, and trait mindfulness measures as secondary predictors to the mixed models and found inconclusive results with regard to HR. While higher maternal and paternal care predicted lower HR during the video segment in Study 1, we found no significant influence of parental care or overprotection on HR in Study 2. This speaks perhaps for a smaller effect, however our statistical power and hence our chances to detect a smaller effect were actually improved in Study 2 with a larger sample size. Results appear more consistent for RSA and measures of early life adversity. In Study 1, higher paternal care predicted higher RSA before and after but not during the video. In Study 2, maternal care was related to higher RSA, while maternal overprotection was associated with lower RSA in response to the video. In addition, in Study 2, higher paternal overprotection predicted lower RSA independent of time.

While the link between HRV and psychopathology, e.g., in post-traumatic stress disorder or depression, is well established (Beauchaine and Thayer, 2015), the discussion to which extent early life adversity, particularly careless or overprotective parenting, in particular, contribute to autonomic dysregulations or an overall lower HRV is still ongoing. For example, lower resting HRV was predicted by higher inconsistent discipline, corporal punishment, and lower parental involvement (Graham et al., 2017) and a blunted HR stress response is linked to the experience of early life adversity (Voellmin et al., 2015).

It is important to consider the various means of observing HRV. While many studies look at RSA at baseline, or resting condition, there is also a number of studies who have looked at RSA changes in response to stress. These have not shown systematic effects, however. For example, RSA change in reaction to the Trier Social Stress Test (Busso et al., 2017) could not be associated with experiences of interpersonal violence or poverty. Similarly, in healthy women with adverse childhood experiences RSA did not mediate the physiological reaction to the Montreal Imaging Stress Task (Winzeler et al., 2017). It is conceivable that a stressor is not the right experimental manipulation to observe systematic associations between the parasympathetic nervous system and variables of personality, or early life adversity. The parasympathetic system is not being stimulated by a stressful situation, but will withdraw; thus, in these cases the experiment will investigate not whether the system can be activated, but if it can be shut down. The null findings in these studies could be a consequence of this distinction.

Our results support the findings that lower parental care and higher parental overprotection might lead to parasympathetic dysregulation that could be associated with psychopathological development later in life (Meyer et al., 2016). Especially the observation that higher paternal overprotection was associated with an overall lower HRV sheds light on the often-overlooked role of fathers’ parenting behavior in psychophysiological development. For example, it was found that fathers appropriate mind-related comments (e.g., “validation of infant’s internal state”) increased the baseline HRV of their one-year old child, independent of the mothers influence (Zeegers et al., 2018). In other aspects the influence of fathers’ parenting behavior on children is similar to the influence of mothers parenting, for example, the same behaviors promote secure attachment (Fagan et al., 2014). Additionally, the effect of mothers and fathers parenting style on their children influence each other (Fagan et al., 2014). Because fathers influence is less well understood and researched as mothers influence (Unternaehrer et al., 2021), it seems highly recommended to include both aspects of parenting in our research on the influence of early life adversity and parenting behavior.

On the other hand, for trait mindfulness, again mixed findings were observed: contrary to our expectations, we found higher scores on the observing subscale (indicating better mindful observing skills) to predict higher HR and lower RSA during the video in Study 1 only. This finding that higher scores of trait mindfulness, especially mindful observing skills, are associated with higher HR and lower RSA seems counterintuitive at first since it suggests less relaxation during the video. We assessed trait mindfulness using the FFMQ, including observing as one subscale, which can be seen as a cognitive function linked to attention. It was found that mindfulness positively influences executive control (Verhaeghen, 2021), self-regulation (Tang and Posner, 2009), and attention (Jha et al., 2007; Tang and Posner, 2009; Verhaeghen, 2021). This link between mindfulness and cognitive functions in general, and attention in particular, might explain why parasympathetic activity was lower during the videos for participants who scored high on the FFMQ-observing subscale. When a cognitive challenge, like focusing on something, arises, it decreases parasympathetic activity and, therefore, HRV (Giuliano et al., 2018). Thus, it is possible that scoring high on mindful observing skills goes along with increased attention to one’s surroundings and internal processes. This association between mindful observing and cognitive effort could affect innervation of the parasympathetic nervous system. Additionally, higher FFMQ scores are associated with better performance in sustained attention (Rice and Liu, 2017) and higher flexibility in changing one’s attention focus, for example directing attention from unimportant stimuli to those needed to accomplish a task (Sørensen et al., 2018). These links suggest that mindfulness contributes to the ability to willingly direct attention, which can be interpreted as voluntary attention, as mentioned in the *Attention Restoration Theory* (Kaplan, 1995). According to this theory, voluntary attention stands opposite to involuntary attention, which is needed to achieve restoration. Therefore, it could be concluded that the aspects of mindfulness focusing on voluntary directing attention counteract the achievement of restoration. This counteraction could inhibit relaxation and therefore explain the low HRV while viewing the videos. In line with this argumentation, Watford and colleagues reported overall lower HF-HRV in persons scoring higher on a trait mindfulness questionnaire. These results seemed to be linked to heightened vigilance and cognitive load in more mindful participants (Watford et al., 2020). In our data, this effect was only visible in Study 1 and could not be replicated in Study 2 and should thus be interpreted with caution.

The relationship between HRV and mindfulness experience is a complex one. We found that less mindfulness experience is associated with a lower HRV in general but scoring high on trait mindful observing skills leads to reduced HRV during the video. It is important to note that there is a fundamental difference between resting-state HRV and HRV in reaction to a stimulus. Therefore, it is not counterintuitive that mindfulness skills might lead to an increase in baseline HRV but prohibit a strong increase in reaction to a relaxation video.

### Limitations and Future Research

Several limitations should be considered when interpreting these results. First and foremost, we planned Study 1 with more liberal exclusion criteria and included a questionnaire baseline only. Adding a physiological baseline (Laborde, 2017) in Study 2 allowed for a more rigorous design but at the same time limited comparability between the two studies. Since the baseline measure of Study 1 was taken while participants filled out questionnaires it is possible that this activity influenced the HRV, making it difficult to compare the baseline levels of HR and HRV with the video response. In Study 2, however, neither HR nor HRV changed significantly between the physiological baseline and the first paper-pencil questionnaire segment. Nevertheless, it was not possible to combine data from Study 1 and Study 2. Thus, sample size might have been too small to detect small effects for secondary predictors and replications in bigger samples are needed to investigate the influence of different psychological characteristics like early life adversity or trait mindfulness on autonomic regulation.

Second, as discussed previously, the selected video material might not have been appropriate to investigate differential effects of a nature videos for several reasons: (a) content of the control video, (b) video presentation on laptop screens, and (c) unbalanced nature sceneries in the nature video condition. The control video, as discussed before, might have been too similar to the experimental condition and thus, we failed to investigate group differences even though we attempted to create comparable control intervention with the movie clip. Many studies researching the relaxing effects of a natural environment have used urban environments to search for group differences. This can be seen in real-life experiments, for example, the effect of a walk in a forest compared to a walk through a city (Song et al., 2018). The forest walk shows a positive effect on anxiety and negative affect. Some experimenters used virtual representations of nature and urban environments to compare their effects on physiological and psychological parameters (Gladwell et al., 2012; Tang et al., 2017). Intervention material displaying urban environments in comparison to nature environments might thus be better suited for this line of research. Furthermore all videos were presented on rather small laptop screens (17-inch in Study 1 and 13-inch in Study 2). As we cannot exclude the possibility that screen size might have influenced relaxation effect, a more systematic investigation of effects of presentation mode (e.g., screen size and resolution, seating distance, acoustic properties) would be helpful for future studies. Moreover, we abstained from analyzing differential effects of nature video content (e.g., forest vs. fireplace) due to unbalanced groups even though. As soundscape studies have revealed, not only visual but also acoustic characteristics of rural videos elicit a psycho-physiological relaxation effect (e.g., subjective ratings as pleasant and restorative, HR decrease) with differential effects, e.g., of water sound compared to bird songs (Ratcliffe, 2021). While we werent́ able to compare different video characteristics within the nature video condition, future research should focus on this particular comparison or determine only one scenery at the start, for example “rain falling in the thicket” which was the most prominent video in this study.

The observation of overall lower RSA during the video condition in Study 2 was unexpected, but as this main effect was independent of the video intervention, it is most likely linked to group differences that exist beyond the experimental manipulation of our study. Though we tried to form comparable groups by quasi-randomly assigning participants to the different conditions, a more comprehensive pre-screening might be necessary in future studies to prevent such an effect. This should be applied especially when psychological characteristics that could constitute group differences are part of the research focus.

Fourth, both study samples consisted of relatively young participants, with an age range of 18 to 49 and only *n* = 4 participants older than 30 years. Taking age into account when measuring HRV is especially important since HRV decreases with age in our and other studies, mainly between 20 and 35 years (Hirsch and Bishop, 1981). Therefore, our study findings are limited to young adulthood, and future studies might want to recruit a sample with a wider age range to be able to measure the reaction to the intervention over the whole lifespan. Additionally, the sample consisted of mostly healthy participants. Only in Study 1 were some participants with a BDI-II score above 18 (*n* = 9) which suggests clinically relevant depressive symptoms (Hautzinger et al., 2006). Like age, mental and physical diseases influence HRV (Acharya et al., 2006; Shaffer and Ginsberg, 2017) and should be taken into account. Only adding healthy participants to the sample was necessary to maximize the comparability of the different groups and limit additional factors influencing the HRV. Beyond age and health, various factors influence HRV, which future studies should take into account: for example, the menstrual cycle (Vallejo et al., 2005), sleep (Glos et al., 2014), and smoking (Barutcu et al., 2005). In addition, it would be interesting to not only look at sex effects but take gender effects into account as well. Furthermore, we measured BMI in Study 2 only and cannot draw any conclusions about its influence in the sample of Study 1. The impact of between-subject differences is reflected by the inter-individual variance as indicated by random effects. While this points toward individual factors that might explain additional variance, the control for random effects in the multilevel model approach is a strength of this data-driven approach (Curran et al., 2010), Those factors could be measured to control their influence or a within-subject design could ensure that those factors do not influence the differences between two interventions. Yet, when using a within-subject design, sequence effects must be taken into account.

## Conclusion

The nature videos, the meditation video and the movie clip we used in these two studies, were effective in producing a robust increase in RSA as a standard HRV measure, together with a decrease in HR. This autonomic response, which is in line with a state of relaxation, may be attributed to the restorative properties of the video material. Looking at secondary predictors, we found evidence of an attenuated relaxation response in association with lower parental care and higher parental overprotection as expected. The observing facet of trait mindfulness seemed to be associated with an attenuated autonomic relaxation response as well. Future research could focus on determining which features of video-based nature scenes specifically promote relaxation when engaged in video viewing. Such videos could thus become useful tools for promoting relaxation in clinical and therapeutic setting.

## Data Availability Statement

The datasets presented in this study can be found in online repositories. The names of the repository/repositories and accession number(s) can be found below: https://osf.io/wcdku/.

## Ethics Statement

The studies involving human participants were reviewed and approved by Ethics Committee of the University of Konstanz. The patients/participants provided their written informed consent to participate in this study.

## Author Contributions

AB carried out the project administration, data curation, and formal analysis, did the conceptualization, performed the methodology, investigated the data, visualized the data, wrote the original draft, and edited the manuscript. RG wrote the original draft, edited the manuscript, carried out the formal analysis, and visualized the data. SS, CJ, and MW performed the methodology, collected the data, wrote, reviewed, and edited the manuscript. MM, UB, BD, and SD wrote, reviewed, and edited the manuscript. EU carried out the formal analysis, wrote, reviewed, and edited the manuscript, and performed the methodology. JP carried out the formal analysis and resources, wrote the original draft, supervised the data, did the conceptualization, and performed the methodology. All authors approved the final version.

## Conflict of Interest

The authors declare that the research was conducted in the absence of any commercial or financial relationships that could be construed as a potential conflict of interest.

## Publisher’s Note

All claims expressed in this article are solely those of the authors and do not necessarily represent those of their affiliated organizations, or those of the publisher, the editors and the reviewers. Any product that may be evaluated in this article, or claim that may be made by its manufacturer, is not guaranteed or endorsed by the publisher.

## Footnotes

1
https://pubmed.ncbi.nlm.nih.gov/?term=relaxation%20video&timeline=expanded

2
https://osf.io/wcdku/

## References

[B1] Acharya U. R. Joseph K. P. Kannathal N. Lim C. M. Suri J. S. (2006). Heart rate variability: a review. *Med. Biol. Eng. Comput.* 44 1031–1051. 17111118 10.1007/s11517-006-0119-0

[B2] Altini M. (2013). *Heart Rate Variability Logger [Mobile Application Software].* Available online at: https://itunes.apple.com/us/app/heart-rate-variability-log-ger/id683984776?ls=1&mt=8. (accessed May 2018).

[B3] Anderson A. P. Mayer M. D. Fellows A. M. Cowan D. R. Hegel M. T. Buckey J. C. (2017). Relaxation with immersive natural scenes presented using virtual reality. *Aerosp Med. Hum. Perform.* 88 520–526. 10.3357/AMHP.4747.2017 28539139

[B4] Azam M. A. Katz J. Fashler S. R. Changoor T. Azargive S. Ritvo P. (2015). Heart rate variability is enhanced in controls but not maladaptive perfectionists during brief mindfulness meditation following stress-induction: a stratified-randomized trial. *Int. J. Psychophysiol.* 98 27–34. 10.1016/j.ijpsycho.2015.06.005 26116778

[B5] Baer R. A. Smith G. T. Hopkins J. Krietemeyer J. Toney L. (2006). Using self-report assessment methods to explore facets of mindfulness. *Assessment* 13 27–45. 10.1177/1073191105283504 16443717

[B6] Balu (2017). *4K Relaxing Fireplace with Crackling Fire Sounds [Video file].* Available online at: https://www.youtube.com/watch?v=bmGsQkLb4yg (accessed March 2018).

[B7] Barutcu I. Esen A. M. Kaya D. Turkmen M. Karakaya O. Melek M. (2005). Cigarette smoking and heart rate variability: dynamic influence of parasympathetic and sympathetic maneuvers. *Ann. Noninvasive Electrocardiol.* 10 324–329. 10.1111/j.1542-474X.2005.00636.x 16029383 PMC6932108

[B8] Beauchaine T. P. Thayer J. F. (2015). Heart rate variability as a transdiagnostic biomarker of psychopathology. *Int. J. Psychophysiol.* 98 338–350. 10.1016/j.ijpsycho.2015.08.004 26272488

[B9] Benz A. B. E. Kloker L. V. Kuhlmann T. Meier M. Unternaehrer E. Bentele U. U. (2021). Psychometrische kennwerte einer deutschen übersetzung des parental bonding instrument. *Psychother. Psychosom. Med. Psychol.* 72, 34–44. 10.1055/a-1503-5328 34255328

[B10] Bertsch K. Hagemann D. Naumann E. Schächinger H. Schulz A. (2012). Stability of heart rate variability indices reflecting parasympathetic activity. *Psychophysiology* 49 672–682. 10.1111/j.1469-8986.2011.01341.x 22335779

[B11] Beute F. De Kort Y. A. W. (2014). Natural resistance: Exposure to nature and self-regulation, mood, and physiology after ego-depletion. *J. Environ. Psychol.* 40 167–178.

[B12] Bhasin M. K. Dusek J. A. Chang B.-H. Joseph M. G. Denninger J. W. Fricchione G. L. (2013). Relaxation Response Induces Temporal Transcriptome Changes in Energy Metabolism. Insulin Secretion and Inflammatory Pathways. *PLoS One* 8:e62817. 10.1371/journal.pone.0062817 23650531 PMC3641112

[B13] Brown D. K. Barton J. L. Gladwell V. F. (2013). Viewing nature scenes positively affects recovery of autonomic function following acute-mental stress. *Environ. Sci. Technol.* 47 5562–5569. 10.1021/es305019p 23590163 PMC3699874

[B14] Brown K. W. Ryan R. M. (2003). The benefits of being present: mindfulness and its role in psychological well-being. *J. Pers. Soc. Psychol.* 84:822. 10.1037/0022-3514.84.4.822 12703651

[B15] Browning M. H. Mimnaugh K. J. Van Riper C. J. Laurent H. K. LaValle S. M. (2020). Can simulated nature support mental health? Comparing short, single-doses of 360-degree nature videos in virtual reality with the outdoors. *Front. Psychol.* 10:2667. 10.3389/fpsyg.2019.02667 32010003 PMC6974516

[B16] Burg J. M. Wolf O. T. Michalak J. (2012). Mindfulness as self-regulated attention: associations with heart rate variability. *Swiss J. Psychol.* 71 135–139. 10.1024/1421-0185/a000080

[B17] Busso D. S. McLaughlin K. A. Sheridan M. A. (2017). Dimensions of adversity, physiological reactivity, and externalizing psychopathology in adolescence: deprivation and threat. *Psychosom. Med.* 79:162. 10.1097/PSY.0000000000000369 27428857 PMC5237627

[B18] Chang B.-H. Dusek J. A. Benson H. (2011). Psychobiological Changes from Relaxation Response Elicitation: Long-Term Practitioners vs. Novices. *Psychosomatics* 52 550–559. 10.1016/j.psym.2011.05.001 22054625

[B19] Curran P. J. Obeidat K. Losardo D. (2010). Twelve frequently asked questions about growth curve modeling. *J. Cogn. Dev.* 11 121–136. 10.1080/15248371003699969 21743795 PMC3131138

[B20] Dana LynnC. (2014). Hearth and campfire influences on arterial blood pressure: defraying the costs of the social brain through fireside relaxation. *Evol. Psychol.* 12 983–1003. 10.1177/14747049140120050925387270

[B21] Engert V. Buss C. Khalili-Mahani N. Wadiwalla M. Dedovic K. Pruessner J. C. (2010). Investigating the association between early life parental care and stress responsivity in adulthood. *Dev. Neuropsychol.* 35 570–581. 10.1080/875656412010494752 20721776

[B22] Fagan J. Day R. Lamb M. E. Cabrera N. J. (2014). Should researchers conceptualize differently the dimensions of parenting for fathers and mothers? *J. Fam. Theory Rev.* 6 390–405.

[B23] Faul F. Erdfelder E. Lang A.-G. Buchner A. (2007). G*Power 3: A flexible statistical power analysis program for the social, behavioral, and biomedical sciences. *Behav. Res. Methods* 39 175–191. 10.3758/BF03193146 17695343

[B24] García Martínez C. A. Otero Quintana A. Vila X. A. Lado Touriño M. J. Rodríguez-Liñares L. Rodríguez Presedo J. M. (2017). *Heart Rate Variability Analysis with the R Package RHRV.* New York, NY: Springer International Publishing, 10.1007/978-3-319-65355-6

[B25] Giuliano R. J. Karns C. M. Bell T. A. Petersen S. Skowron E. A. Neville H. J. (2018). Parasympathetic and sympathetic activity are associated with individual differences in neural indices of selective attention in adults. *Psychophysiology* 55:e13079. 10.1111/psyp.13079 29624675 PMC12634002

[B26] Gladwell V. F. Brown D. K. Barton J. L. Tarvainen M. P. Kuoppa P. Pretty J. (2012). The effects of views of nature on autonomic control. *Eur. J. Appl. Physiol.* 112 3379–3386. 10.1007/s00421-012-2318-8 22270487

[B27] Glos M. Fietze I. Blau A. Baumann G. Penzel T. (2014). Cardiac autonomic modulation and sleepiness: physiological consequences of sleep deprivation due to 40 h of prolonged wakefulness. *Physiol. Behav.* 125 45–53. 10.1016/j.physbeh.2013.11.011 24291386

[B28] Gould C. E. Kok B. C. Ma V. K. Wetherell J. L. Sudheimer K. Beaudreau S. A. (2019). Video-delivered relaxation intervention reduces late-life anxiety: a pilot randomized controlled trial. *Am. J. Geriatr. Psychiatry* 27 514–525. 10.1016/j.jagp.2018.12.018 30765288 PMC13537017

[B29] Graham R. A. Scott B. G. Weems C. F. (2017). Parenting behaviors, parent heart rate variability, and their associations with adolescent heart rate variability. *J. Youth Adolesc.* 46 1089–1103. 10.1007/s10964-016-0616-x 27904984

[B30] Graubner B. (2013). *ICD-10-GM 2014 Systematisches Verzeichnis: Internationale statistische Klassifikation der Krankheiten und verwandter Gesundheitsprobleme 11. Revision-German Modification* Version 2014. Köln: Deutscher Ärzteverlag.

[B31] Hautzinger M. Keller F. Kühner C. (2006). *BDI II Beck Depressions-Inventar Revision.* London: Pearson.

[B32] Heinzen E. Sinnwell J. Atkinson E. Gunderson T. Dougherty G. (2021). *Arsenal: An Arsenal of “R” Functions for Large-Scale Statistical Summaries.* R package version 3.6.2. Available online at: https://CRAN.R-project.org/package=arsenal (accessed May 2021).

[B33] Hirsch J. A. Bishop B. (1981). Respiratory sinus arrhythmia in humans: How breathing pattern modulates heart rate. *Am. J. Physiol.* 241 H620–H629. 10.1152/ajpheart.1981.241.4.H620 7315987

[B34] Holzman J. B. Bridgett D. J. (2017). Heart rate variability indices as bio-markers of top-down self-regulatory mechanisms: a meta-analytic review. *Neurosci. Biobehav. Rev.* 74 233–255. 10.1016/j.neubiorev.2016.12.032 28057463

[B35] Jackson P. (2001). *Der Herr der Ringe: Die Gefährten [Video file].* Available online at: https://itunes.apple.com/de/movie/der-herr-der-ringe-die-gefährten-special-extended-edition/id430796548?ign-mpt=uo%3D4 (accessed July 2018).

[B36] Jain S. Shapiro S. L. Swanick S. Roesch S. C. Mills P. J. Bell I. (2007). A randomized controlled trial of mindfulness meditation versus relaxation training: Effects on distress, positive states of mind, rumination, and distraction. *Ann. Behav. Med.* 33 11–21. 10.1207/s15324796abm3301_2 17291166

[B37] Janicke S. H. Rieger D. Reinecke L. Connor W. (2018). Watching online videos at work: the role of positive and meaningful affect for recovery experiences and well-being at the workplace. *Mass Commun. Soc.* 21 345–367.

[B38] Jha A. P. Krompinger J. Baime M. J. (2007). Mindfulness training modifies subsystems of attention. *Cogn. Affect. Behav. Neurosci.* 7 109–119. 10.3758/cabn.7.2.109 17672382

[B39] Jo H. Song C. Miyazaki Y. (2019). Physiological benefits of viewing nature: a systematic review of indoor experiments. *Int. J. Environ. Res. Public Health* 16:4739. 10.3390/ijerph16234739 31783531 PMC6926748

[B40] Kaplan S. (1995). The restorative benefits of nature: toward an integrative framework. *J. Environ. Psychol.* 15 169–182. 10.1016/0272-4944(95)90001-2

[B41] Kellert S. R. Wilson E. O. (1993). *The Biophilia Hypothesis.* Washington, D.C: Island Press.

[B42] Kemp A. H. Quintana D. S. Gray M. A. Felmingham K. L. Brown K. Gatt J. M. (2010). Impact of depression and antidepressant treatment on heart rate variability: a review and meta-analysis. *Biol. Psychiatry* 67 1067–1074. 10.1016/j.biopsych.2009.12.012 20138254

[B43] Kreibig S. D. (2010). Autonomic nervous system activity in emotion: a review. *Biol. Pschol.* 84 394–421. 10.1016/j.biopsycho.2010.03.010 20371374

[B44] Laborde S. (2017). Heart rate variability and cardiac vagal tone in psychophysiological research – recommendations for experiment planning, data analysis, and data reporting. *Front. Psychol.* 8:18. 10.3389/fpsyg.2017.00213 28265249 PMC5316555

[B45] Lawson J. (2013). *Entspannende Naturgeräusche [Video file].* Available online at: https://www.youtube.com/watch?v=eKFTSSKCzWA (accessed March 2018).

[B46] Lee J. Tsunetsugu Y. Takayama N. Park B.-J. Li Q. Song C. (2014). Influence of forest therapy on cardiovascular relaxation in young adults. *Evid.-Based Complement. Altern. Med.* 2014:834360. 10.1155/2014/834360 24660018 PMC3934621

[B47] Lima A. R. Mello M. F. Mari J. de J. (2010). The role of early parental bonding in the development of psychiatric symptoms in adulthood. *Curr. Opin. Psychiatry* 23 383–387. 10.1097/YCO.0b013e32833a51ce 20540179

[B48] Lüdecke D. Ben-Shachar M. S. Patil I. Waggoner P. Makowski D. (2021). Performance: an r package for assessment, comparison and testing of statistical models. *J. Open Source Softw.* 6:3139. 10.21105/joss.03139

[B49] Luecken L. J. (2000). Parental caring and loss during childhood and adult cortisol responses to stress. *Psychol. Health* 15 841–851. 10.1080/08870440008405586

[B50] Lutz R. Heyn C. Kommer D. (1995). “Fragebogen zur elterlichen Bindung,” in *Wie gesund sind Kranke? Zur seelischen Gesundheit psychisch Kranker*, ed. Lutz R. (Göttingen:Verlag für Angewandt Psychologie).

[B51] Matsui T. Kakisaka K. Shinba T. (2016). Impaired parasympathetic augmentation under relaxation in patients with depression as assessed by a novel non-contact microwave radar system. *J. Med. Eng. Technol.* 40 15–19. 10.3109/03091902.2015.1116632 26728780

[B52] Meier M. Unternaehrer E. Dimitroff S. J. Benz A. B. E. Bentele U. U. Schorpp S. M. (2020). Standardized massage interventions as protocols for the induction of psychophysiological relaxation in the laboratory: a block randomized, controlled trial. *Sci. Rep.* 10:14774. 10.1038/s41598-020-71173-w 32901072 PMC7479151

[B53] Meyer P.-W. Müller L. E. Zastrow A. Schmidinger I. Bohus M. Herpertz S. C. (2016). Heart rate variability in patients with post-traumatic stress disorder or borderline personality disorder: relationship to early life maltreatment. *J. Neural Transm.* 123 1107–1118. 10.1007/s00702-016-1584-8 27311838

[B54] Michalak J. Zarbock G. Drews M. Otto D. Mertens D. Ströhle G. (2016). Erfassung von Achtsamkeit mit der deutschen Version des Five Facet Mindfulness Questionnaires (FFMQ-D). *Z. Gesundheitspsychol.* 24 1–12. 10.1026/0943-8149/a000149

[B55] Morrison M. (2017). *Geführte Anfänger Meditation: 10 Minuten für jeden Tag [Video file].* Available online at: https://www.youtube.com/watch?v=ockCQMt9kM0 (accessed July 2018).

[B56] Outstanding Videos. (2012). *Relaxing 3 Hour Video of A Tropical Beach with Blue Sky White Sand and Palm Tree [Video file].* Available online at: https://www.youtube.com/watch?v=qREKP9oijWI (accessed March 2018).

[B57] Parker G. Tupling H. Brown L. B. (1979). A parental bonding instrument. *Br. J. Med. Psychol.* 52 1–10.

[B58] Paunchev P. (2017). *4K Tropical Rain & Relaxing Nature Sounds [Video file].* Available online at: https://www.youtube.com/watch?v=c9pQYOGIWM8 (accessed March 2018).

[B59] Payne M. D. Delphinus E. (2019). A review of the current evidence for the health benefits derived from forest bathing. *Int. J. Health Wellness Soc.* 9 19–30.

[B60] Peyser D. Scolnick B. Hildebrandt T. Taylor J. A. (2021). Heart rate variability as a biomarker for anorexia nervosa: a review. *Eur. Eat. Dis. Rev.* 29 20–31. 10.1002/erv.2791 32975349

[B61] Pinheiro J. Bates D. DebRoy S. Sarkar D. R Core Team (2021). *_nlme: Linear and Nonlinear Mixed Effects Models_.* R package version 3.1-152. Available online at: https://CRAN.R-project.org/package=nlme (accessed May 2021).

[B62] R Core Team, (2021). *R: A Language and Environment for Statistical Computing. R Foundation for Statistical Computing*. Available online at: https://www.R-project.org/ (accessed May 2021).

[B63] Ratcliffe E. (2021). Sound and soundscape in restorative natural environments: a narrative literature review. *Front. Psychol.* 12:570563. 10.3389/fpsyg.2021.570563 33981262 PMC8107214

[B64] Rice V. J. Liu B. (2017). “The relationship between sustained attention and mindfulness among U.S. active duty service members and veterans,” in *Advances in Social & Occupational Ergonomics*, 487 ed. Goossens R. H. M. (New York, NY: Springer International Publishing), 397–407. 10.1007/978-3-319-41688-5_37

[B65] RStudio Team (2016). *RStudio: Integrated Development Environment for R*. Boston, MA: RStudio, PBC.

[B66] Scott E. E. LoTemplio S. B. McDonnell A. S. McNay G. D. Greenberg K. McKinney T. (2021). The autonomic nervous system in its natural environment: Immersion in nature is associated with changes in heart rate and heart rate variability. *Psychophysiology* 58 e13698. 10.1111/psyp.13698 33048361

[B67] Shaffer F. Ginsberg J. P. (2017). An overview of heart rate variability metrics and norms. *Front. Public Health* 5:258. 10.3389/fpubh.2017.00258 29034226 PMC5624990

[B68] Shonkoff J. P. Garner A. S. The Committee On Psychosocial Aspects Of Child And Family Health, Committee On Early Childhood, Adoption, And Dependent Care, and Section On Developmental And Behavioral Pediatrics (2012). The Lifelong Effects of Early Childhood adversity and toxic stress. *Pediatrics* 129:e232-46. 10.1542/peds.2011-2663 22201156

[B69] Sigrist C. Mürner-Lavanchy I. Peschel S. K. Schmidt S. J. Kaess M. Koenig J. (2020). Early life maltreatment and resting-state heart rate variability: a systematic review and meta-analysis. *Neurosci. Biobehav. Rev.* 120 307–334. 10.1016/j.neubiorev.2020.10.026 33171141

[B70] Smith J. C. (2007). The new psychology of relaxation and renewal. *Biofeedback* 35 85–89.

[B71] Smith K. E. Pollak S. D. (2021). Rethinking concepts and categories for understanding the neurodevelopmental effects of childhood adversity. *Perspect. Psychol. Sci.* 16 67–93. 10.1177/1745691620920725 32668190 PMC7809338

[B72] Song C. Igarashi M. Ikei H. Miyazaki Y. (2017). Physiological effects of viewing fresh red roses. *Complement. Therap. Med.* 35 78–84. 10.1016/j.ctim.2017.10.001 29154072

[B73] Song C. Ikei H. Park B.-J. Lee J. Kagawa T. Miyazaki Y. (2018). Psychological benefits of walking through forest areas. *Int. J. Environ. Res. Public Health* 15:2804. 10.3390/ijerph15122804 PMC6313311 30544682

[B74] Sørensen L. Osnes B. Visted E. Svendsen J. L. Adolfsdottir S. Binder P.-E. (2018). Dispositional mindfulness and attentional control: the specific association between the mindfulness facets of non-judgment and describing with flexibility of early operating orienting in conflict detection. *Front. Psychol.* 9:2359. 10.3389/fpsyg.2018.02359 30555383 PMC6282922

[B75] Strüven A. Holzapfel C. Stremmel C. Brunner S. (2021). Obesity, nutrition and heart rate variability. *Int. J. Mol. Sci.* 22:4215. 10.3390/ijms22084215 33921697 PMC8072942

[B76] Tang I.-C. Tsai Y.-P. Lin Y.-J. Chen J.-H. Hsieh C.-H. Hung S.-H. (2017). Using functional Magnetic Resonance Imaging (fMRI) to analyze brain region activity when viewing landscapes. *Landsc. Urban Plan.* 162 137–144. 10.1016/j.landurbplan.2017.02.007

[B77] Tang Y.-Y. Posner M. I. (2009). Attention training and attention state training. *Trends in Cogn. Sci.* 13 222–227. 19375975 10.1016/j.tics.2009.01.009

[B78] Tarullo A. R. Gunnar M. R. (2006). Child maltreatment and the developing HPA axis. *Horm. Behav.* 50 632–639. 10.1016/j.yhbeh.2006.06.010 16876168

[B79] Thayer J. F. Åhs F. Fredrikson M. Sollers J. J. Wager T. D. (2012). A meta-analysis of heart rate variability and neuroimaging studies: implications for heart rate variability as a marker of stress and health. *Neurosci. Biobehav. Rev.* 36 747–756. 10.1016/j.neubiorev.2011.11.009 22178086

[B80] Ulrich R. S. (1983). “Aesthetic and affective response to natural environment,” in *Behavior and the Natural Environment*, eds Altman I. Wohlwill J. F. (New York, NY: Springer), 85–125. 10.1007/978-1-4613-3539-9_4

[B81] Unternaehrer E. Meier M. Bouvette-Turcot A.-A. Hari Dass S. A. (2021). “Long-term epigenetic effects of parental caregiving,” in *Developmental Human Behavioral Epigenetics*, eds Provenzi L. Montirosso R. (Amsterdam: Elsevier), 105–117. 10.1016/B978-0-12-819262-7.00006-4

[B82] Vallejo M. Márquez M. F. Borja-Aburto V. H. Cárdenas M. Hermosillo A. G. (2005). Age, body mass index, and menstrual cycle influence young women’s heart rate variability. *Clin. Auton. Res.* 15 292–298. 10.1007/s10286-005-0272-9 16032384

[B83] Van den Berg M. M. Maas J. Muller R. Braun A. Kaandorp W. Van Lien R. (2015). Autonomic nervous system responses to viewing green and built settings: Differentiating between sympathetic and parasympathetic activity. *Int. J. Environ. Res. Public Health* 12 15860–15874. 10.3390/ijerph121215026 26694426 PMC4690962

[B84] Verhaeghen P. (2021). Mindfulness as attention training: Meta-analyses on the links between attention performance and mindfulness interventions, long-term meditation practice, and trait mindfulness. *Mindfulness* 12 564–581.

[B85] Voellmin A. Winzeler K. Hug E. Wilhelm F. H. Schaefer V. Gaab J. (2015). Blunted endocrine and cardiovascular reactivity in young healthy women reporting a history of childhood adversity. *Psychoneuroendocrinology* 51 58–67. 10.1016/j.psyneuen.2014.09.008 25290347

[B86] Watford T. S. O’Brien W. H. Koerten H. R. Bogusch L. M. Moeller M. T. Singh R. S. (2020). The mindful attention and awareness scale is associated with lower levels of high-frequency heart rate variability in a laboratory context. *Psychophysiology* 57:e13506. 10.1111/psyp.13506 31737916

[B87] Wickham H. (2016). *ggplot2: Elegant Graphics for Data Analysis.* Berlin: Springer-Verlag.

[B88] Winzeler K. Voellmin A. Hug E. Kirmse U. Helmig S. Princip M. (2017). Adverse childhood experiences and autonomic regulation in response to acute stress: the role of the sympathetic and parasympathetic nervous systems. *Anxiety Stress Coping* 30 145–154. 10.1080/10615806.2016.1238076 27653030

[B89] Yu C.-P. Lin C.-M. Tsai M.-J. Tsai Y.-C. Chen C.-Y. (2017). Effects of short forest bathing program on autonomic nervous system activity and mood states in middle-aged and elderly individuals. *Int. J. Environ. Res. Public Health* 14:897. 10.3390/ijerph14080897 28792445 PMC5579495

[B90] Yu D. S. Lee D. T. Woo J. (2010). Improving health-related quality of life of patients with chronic heart failure: Effects of relaxation therapy. *J. Adv. Nurs.* 66 392–403. 10.1111/j.1365-2648.2009.05198.x 20423422

[B91] Zeegers M. A. de Vente W. Nikolić M. Majdandžić M. Bögels S. M. Colonnesi C. (2018). Mothers’ and fathers’ mind-mindedness influences physiological emotion regulation of infants across the first year of life. *Dev. Sci.* 21:e12689. 10.1111/desc.12689 29920863 PMC6220880

[B92] Zeidan F. Johnson S. K. Diamond B. J. David Z. Goolkasian P. (2010). Mindfulness meditation improves cognition: evidence of brief mental training. *Conscious. Cogn.* 19 597–605. 10.1016/j.concog.2010.03.014 20363650

